# Occipital posterior interhemispheric supratentorial approach for resection of midbrain cavernous malformation

**DOI:** 10.3171/2021.4.FOCVID2133

**Published:** 2021-07-01

**Authors:** Turki Elarjani, Nickalus R. Khan, Samir Sur, Jacques J. Morcos

**Affiliations:** 1 Department of Neurological Surgery, University of Miami, Florida; and; 2 Department of Neurosurgery, Georgetown University, Washington, DC

**Keywords:** pineal gland, cavernous malformation, occipital posterior interhemispheric supratentorial approach

## Abstract

Approaches to the pineal region are various, and each has its advantages and disadvantages. The authors present a case of a 50-year-old woman who presented with progressive hemiparesis and vertical gaze palsy; she was diagnosed with a midbrain cavernous malformation. The patient underwent an occipital posterior interhemispheric supratentorial transpineal approach with gross-total resection. On long-term follow-up, her symptoms significantly improved. The authors review the regional anatomy and present the operative video. They also discuss the various approaches with their indications, advantages, and disadvantages.

The video can be found here: https://stream.cadmore.media/r10.3171/2021.4.FOCVID2133.

## Transcript

### 0:30 Clinical Presentation

This is a 50-year-old female diagnosed with a midbrain cavernous malformation who underwent radiosurgery at an outside institution. Her symptoms progressed into near-complete bilateral vertical gaze palsy, right hemiparesis, and dysarthria. She was referred to our institution for further management. Her neurological examination included a complete vertical gaze palsy, 4/5 power in her right upper and lower extremity, as well as hyperreflexia on the right.

### 1:06 Neuroimaging Findings

This T2 sequence MRI shows a classic appearance of cavernous malformation with a hemosiderin ring visualized. Here is a T1 coronal and sagittal image showing the cavernous malformation.

### 1:27 Rationale, Risks, Benefits, and Steps of the Procedure

The rationale for the procedure is for the progressive neurological deficits and prevention of mass effect and hemorrhage. The risks of the procedure are listed below, and the benefits are the prevention of a rehemorrhage and to remove the mass effect. There are no good alternatives for treatment. The setup included a Mayfield head holder and a supine position with the head rotated toward the right with a posterior midline incision. The microscope, as well as intraoperative neuronavigation and neuromonitoring, were used. The key surgical steps are listed below.

### 2:12 Approach and Craniotomy Location With an Anatomical Correlation

The approach of choice was an occipital posterior interhemispheric supratentorial approach. This cartoon shows the planned craniotomy site, which extends across the sagittal sinus. This is a Rhoton image illustrating the regional anatomy including the relationship of the pineal gland, third ventricle, tectal plate, fourth nerve, and the tentorium.

### 2:45 Interhemispheric Approach

Here we are with the craniotomy performed. The right side is down with gravity retraction. This is the beginning phase of the interhemispheric dissection. Neuronavigation is used to identify the location of the straight sinus. This Rhoton image shows the relationship of the straight sinus, third ventricle, cavernous malformation, and the pineal gland.

### 3:26 Dividing the Tentorium

We now prepare to divide the tentorium. One can appreciate the retractorless surgical corridor provided by the right side of the head being down with gravity-assisted retraction. Microsurgical techniques are used to visualize both sides of the tentorium to prevent neurovascular injury. The tentorium is now divided to widen the surgical corridor.

### 4:13 Arachnoid Dissection Over the Deep Venous System

Since the surgical corridor was widened, we can visualize the deep venous system, which includes the vein of Galen, Rosenthal, internal cerebral vein, and the pineal gland. This view is more posterior, and our approach came from a slightly more posterolateral trajectory. The arachnoid over the vein of Galen is visualized and divided. The pineal gland now comes into view. One can appreciate the splenium of the corpus callosum.

### 5:03 Transpineal Approach and Resection of the Cavernous Malformation

The pineal gland is now transgressed. The pineal gland has now been removed and the cavernous malformation is well visualized. Throughout this process, neuronavigation was used in order to confirm our trajectory was appropriate. The resection of the cavernous malformation now begins using standard microsurgical techniques. SSEP and MEP, as well as cranial nerve monitoring, was utilized throughout this resection. One can appreciate the direct access to this lesion provided by the occipital interhemispheric approach. The cavernous malformation is now removed. The surgical cavity is inspected to ensure no residual remains.

### 6:49 Disease Background and Review of Different Approaches to the Pineal Region

Cavernous malformations are low-flow lesions.^1^ Brainstem cavernous malformations comprise 9%–35% of cavernous malformations, with hemorrhage rates listed below.^2,3^ We chose the occipital posterior interhemispheric supratentorial approach because it offers a direct access to the lesion, given the fact that a steep tentorium was present.^4–6^ The supracerebellar infratentorial approach even with a paramedian extension was not the approach of choice due to the steepness of the tentorium and the superior extension of the cavernous malformation to the third ventricle. The indications, advantages, and disadvantages are listed here.

Other approaches have been described but were not the approach of choice in this situation. The anterior interhemispheric transcallosal approach could access the third ventricle; however, the working corridor would have been much longer and less direct to the lesion. The anterolateral transsylvian approach or endoscopic supracerebellar infratentorial have also been described but were not considered appropriate for this lesion.^7–9^ Their indications, advantages, and disadvantages are listed.

### 8:12 Follow-Up Clinical Exam and Radiological Findings

The patient remained at her neurological baseline in the immediate postoperative period and showed significant improvement in her right upper and lower extremity and vertical gaze palsy on long-term follow-up. The postoperative MRI showed gross-total resection of the cavernous malformation. Our references are listed here.

## Author Contributions

Primary surgeon: Morcos. Assistant surgeon: Sur. Editing and drafting the video and abstract: Morcos, Elarjani, Khan, Sur. Critically revising the work: Morcos, Elarjani, Khan. Reviewed submitted version of the work: all authors. Approved the final version of the work on behalf of all authors: Morcos. Supervision: Morcos.
